# Co-infection by multiple vector-borne agents in wild ring-tailed coatis (*Nasua nasua*) from Iguaçu National Park, southern Brazil

**DOI:** 10.1038/s41598-023-29090-1

**Published:** 2023-02-01

**Authors:** L. Perles, M. F. Moraes, M. Xavier da Silva, R. F. C. Vieira, R. Z. Machado, E. G. Lux Hoppe, M. R. André

**Affiliations:** 1 Vector-Borne Bioagents Laboratory (VBBL), Department of Pathology, Reproduction and One Health, School of Agricultural and Veterinarian Sciences, São Paulo State University (Unesp), Via de Acesso Prof. Paulo Donato Castellane, s/n, Zona Rural, Jaboticabal, São Paulo CEP: 14884-900 Brazil; 2 Laboratory of Parasitic Diseases (LabEPar), Department of Pathology, Reproduction and One Health, School of Agricultural and Veterinarian Sciences, São Paulo State University (Unesp), Jaboticabal, SP Brazil; 3 Iguaçu Carnivore Project, Iguaçu National Park, BR-469, Km 22.5, Foz do Iguaçu, Paraná 85851-970 Brazil; 4 Vector-Borne Diseases Laboratory, Department of Veterinary Medicine, Universidade Federal do Paraná – UFPR, Curitiba, Brazil; 5 Department of Public Health Sciences, University of North Carolina at Charlotte, Charlotte, USA; 6 Center for Computational Intelligence to Predict Health and Environmental Risks (CIPHER), University of North Carolina at Charlotte, Charlotte, USA

**Keywords:** Parasitology, Pathogens

## Abstract

The present study aimed to detect molecularly the presence of co-infections by vector-borne agents (VBA) in ring-tailed coatis’ (*Nasua nasua*) blood samples from Iguaçu National Park (INP), southern Brazil, and assess the phylogenetic positioning of the detected agents. DNA blood samples were submitted to molecular screening and characterization for Anaplasmataceae agents, Piroplasmids, *Hepatozoon* sp., hemotropic mycoplasmas, and *Bartonella* spp. In total, 42 (85.7%) coatis were positive for hemotropic *Mycoplasma* sp., 12 (24.5%) for *Bartonella machadoae*, 7 (14.3%) for *Anaplasma* sp. closely related to ‘*Candidatus* Anaplasma brasiliensis’, and 3 (6%) for *Hepatozoon procyonis*. The most prevalent co-infections observed was from bacterial VBA: while 18.3% were co-infected by hemotropic *Mycoplasma* sp. and *Bartonella* sp., 12.2% were co-infected by *Anaplasma* sp. and hemotropic *Mycoplasma* sp. Only two animals (4%) presented co-infections by three VBA (*Bartonella* sp., *Anaplasma* sp. and hemotropic *Mycoplasma* sp.). The coati is a wild carnivore found in INP, mainly in areas visited by tourists. These animals are frequently seen searching for food in garbage dumps or in tourists’ belongings. The present study expands the host specificity range of *B. machadoae*, which has been isolated only from rodents until the present moment. Since the zoonotic potential and transmission routes of the detected VBA are not yet known, surveillance in this area is much needed.

## Introduction

Ring-tailed coatis (*Nasua nasua* Carnivora: Procyonidae) are widely distributed in South America. They are known to be social animals, forming large familiar groups up to 30 individuals, composed mainly of females in reproductive age and few mature males^1,2^. Coatis adapt very easily in urbanized areas, where they can interact with domestic animals, such as dogs and cats^2,3^. In these environments they are used to explore and feed on anthropogenic food sources (e.g. garbage)^4,5^. The Iguaçu National Park (INP) is one of the largest remaining areas of the Atlantic Forest in Brazil, and is also an important tourist attraction, receiving annually 1.5 million visitors from many regions of the world (https://www.icmbio.gov.br/parnaiguacu/). At INP coatis are known to interact constantly with tourists by locking for food, which eventually results in human injuries^6^. Such mammals can harbor several pathogens, such as rabies virus, *Trypanosoma cruzi* and *Leishmania* spp., zoonotic agents of great importance in Public Health^7,8^.

Vector-borne agents (VBA) are pathogens (e.g. bacteria, virus, protozoa) transmitted by a plethora of vectors, such as ticks, fleas, lice, mosquitoes, sandflies^9,10^. Some agents transmitted by vectors can cause important economic losses and also important impacts in human health^9,10^. Epidemiological dynamics of VBAs are based on complex interactions among vectors, pathogens and vertebrate hosts. Regarding VBAs in coatis from Brazil, hemotropic *Mycoplasma* spp. has been detected in Mato Grosso do Sul and Paraná state^11,12^ and recently, ‘*Candidatus* Mycoplasma haematonasua’ has been described in coatis from “Parque Nacional do Iguaçu” (PARNA Iguaçu)^13^. Regarding Apicomplexan protozoan, *Hepatozoon procyonis* has been widely detected in several Brazilian regions^14–18^, while Piroplasmids agents were detected only in coatis sampled in the Pantanal of Mato Grosso do Sul state^19^ and in Minas Gerais state^17^. Interestingly, Sousa et al.^20^ and Collere et al.^13^ detected Anaplasmataceae agents in blood samples from coatis. While *Ehrlichia* 16S rRNA genotypes closely related to *E. canis/E. chaffeensis,* and *Anaplasma* 16S rRNA genotypes closely related to *A. bovis* and A. *phagocytophilum* on coatis in the Pantanal wetlands, state of Mato Grosso do Sul^20^, Collere et al.^13^ failed to sequence the detected agents. Recently, Perles et al.^21^ described ‘*Candidatus* Ehrlichia dumleri’, a putative novel Anaplasmataceae agent, and *Anaplasma* sp. closely related to ‘C*andidatus* Anaplasma brasiliensis’ in blood samples from coatis in peri-urbanized forested areas in central-western Brazil.

Considering the ecological and zoonotic importance of the knowledge on VBA, the lack of information regarding wild population’s diseases and the methodological complexity of field research with wild animals, the present study aimed to detect molecularly the presence of VBA in coati’s blood samples and assess the phylogenetic positioning of the detected agents. Moreover, the INP is one of the most important Atlantic Forest Protected area, that still harbor most of the terrestrial large mammals and it is also the second most visited National Park in Brazil^22^. To the best of the authors’ knowledge, there is no report of *Bartonella* spp. in coatis from Brazil, highlighting the importance of surveillance of vector-borne agents (VBA) in wild mammals.

## Methods

### Ethical aspects

All methods were carried out in accordance with relevant guidelines and regulations and were approved by the local Ethics Committee on Animal Use of São Paulo State University—Unesp, Agrarian and Veterinarian Sciences School—FCAV (Protocol 07553/14) and by the Brazilian Authorization and Information System—SISBIO (Protocol 38006-2); and “Sistema Nacional de Gestão de Patrimônio Genético e do Conhecimento Tradicional Associado”—SISGEN (Protocol AAA5680); permit SISBIO (numbers: 48.141–5, 62.541–6, 38.006–2, 38.006–4, 38.006–6, and 38.006–8). All methods are reported at the present work are in accordance with ARRIVE guidelines (https://arriveguidelines.org).

### Study area and animal trapping

**Figure 1:**
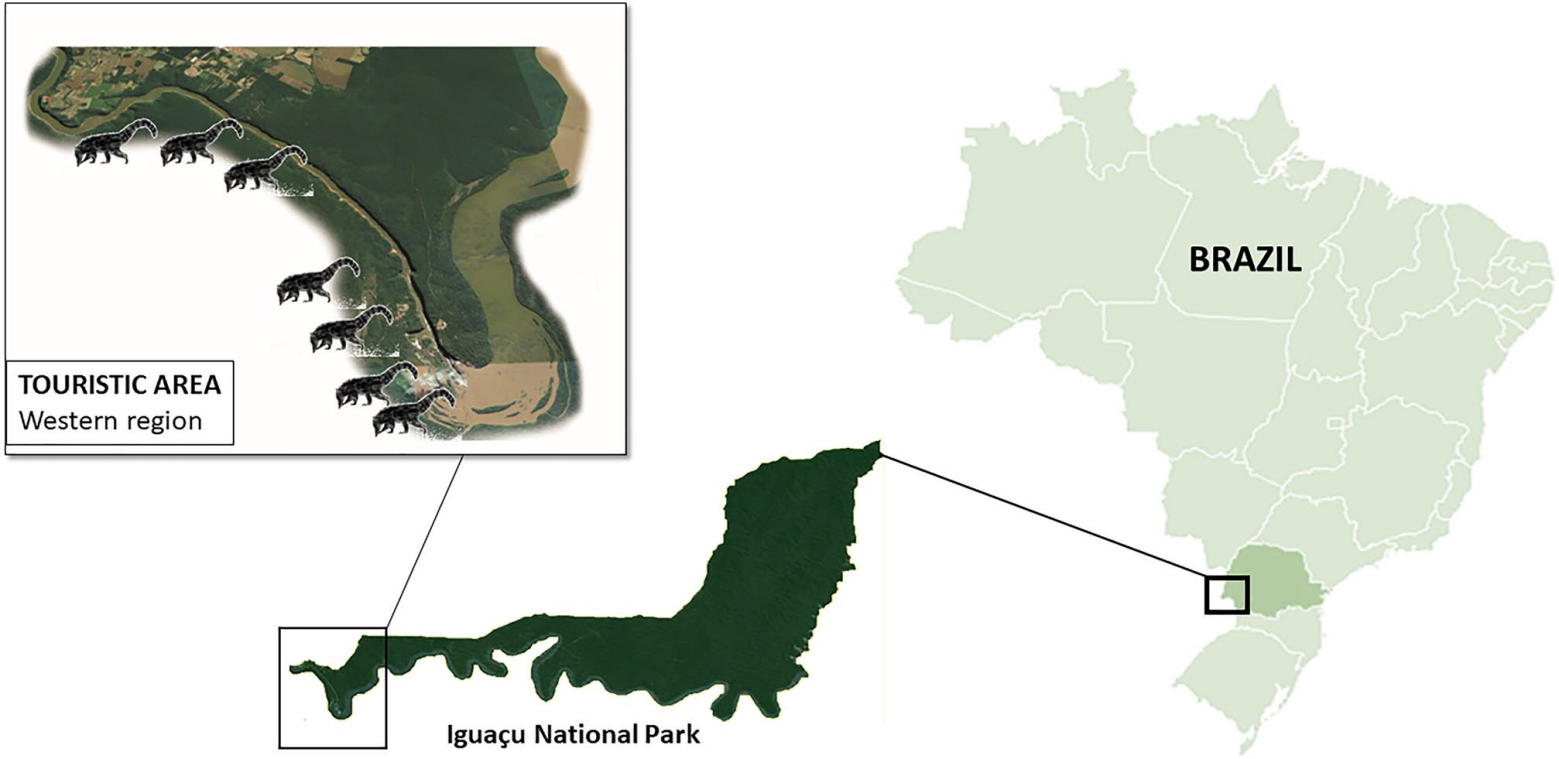
Map showing Paraná State (light green) and Iguaçu National Park (dark green).

PARNA Iguaçu is located within the Atlantic Forest Biome in the western state of Paraná, Brazil, representing one of the last remnants of this important Biome (25°05′ S to 25°40′ S and 54°30′ W to 54°40′ W)^22^ (Fig. 1). The park contains up to 185,000 hectares of semi-deciduous forest and its fauna is highly diverse, with records of 102 species of mammals, 386 birds, 48 reptiles, 13 amphibians, 176 fishes, 839 invertebrates, and 28 species of parasite helminths, most of them endemic to the Atlantic rainforest^22^.

Between August 2014 and December 2017, coatis (*Nasua nasua)* were captured with hand nets and Tomahawk traps baited with a mixture of fresh chicken meat, fruits (banana, pineapple or mango) and peanut butter, during several campaigns, totaling 7200 h capture effort. Captures were performed at the Western region of the INP. Following the captures, the animals were physically restrained, anesthetized with tiletamine-zolazepan association (5 mg/kg, IM), and identified with a small numbered ear tag to prevent recaptures. Blood samples from 49 coatis (21 males; 28 females) were drawn from the jugular vein and then stored in EDTA-coated tubes in isothermal boxes with ice and then were transported to the laboratory for processing^23^.

### DNA extraction from blood samples and conventional PCR (PCR) for mammal glyceraldehyde 3-phosphate dehydrogenase (gadph) endogenous gene

DNA was extracted from 200 μL of blood using Biopur Kit Mini Spin Plus, according to the manufacturer’s instructions. To evaluate the quality of the extracted DNA and avoid false negative results, DNA samples were tested by a conventional PCR targeting the mammal *gapdh* gene^24^. Ultra-pure sterile water (Life Technologies®, Carlsbad, CA, USA) was used as a negative control in PCR assays.

### Molecular assays for vector-borne agents

Coati DNA blood samples positive for *gapdh* gene were submitted to PCR assays for *Anaplasma, Ehrlichia, Neorickettsia, Bartonella*, hemoplasmas, *Hepatozoon*, and Piroplasmids. All agents, target gene, primer sequences, size of the amplicon (bp), thermal cycling conditions and references are shown in Table 1 (and Figure SM1—Supplementary file).

**Table 1:** Molecular assays according to the involved agent, type of PCR, target gene, primer sequences, size of the amplicons (bp), thermal cycling conditions and references, used in the present study for screening and characterizing vector-borne agents DNA in coati’ blood sampled in Iguaçu National Park, Paraná state, southern Brazil.

Agents	Aim	Molecular assay	Primers sequences	Fragment size (bp)	Thermal cycling	Reference
*Anaplasma* spp. (16S rRNA gene) External primers gE3a gE10R Internal primers gE2 gE9f.	Screening	nPCR	5′-CACATGCAAGTCGAACGGATTATTC-3′ 5′-TTCCGTTAAGAAGGATCTAATCTCC′-3′	932	94 °C for 5 min 40 cycles: 94 °C for 30 s, 55 °C for 30 s and 72 °C for 1 min 72 °C for 5 min	^25^
			5′-GGCAGTATTAAAAGCAGCTCCAGG-3′ 5′-AACGGATTATTCTTTATAGCTTGCT-3′	546		
*Anaplasma* spp. (ITS—23S–5S) ITS2F ITS2R	Characterization	cPCR	5′-AGGATCTGACTCTAGTAACGAG-3′ 5′-CTCCCATGTCTTAAGACAAAG-3′	300	94 °C for 2 min, 35 cycles; 94 °C for 30 s, 58 °C for 30 s, 72 °C for 1 min 72 °C for 5 min	^26^
*Anaplasma* spp. (*gltA* gene) External primers F4b R1b EHR-C5136F HER-778R	Characterization	nPCR	5′-CCGGGTTTTATGTCTACTGC-3′ 5′-CGATGACCAAAACCCAT-3′ 5′-TTYATGTCYACTGCTGCKTG-3′ 5′-GCNCCMCCATGMGCTGG-3′	700	95 °C for min; 35 cycles: 95 °C for 30 s, 55 °C for 30 s and 72 °C for 30 s and a final extension 72 °C for 10 min	^27^
*Ehrlichia* spp. (*dsb* gene) dsb-330 dsb-728	Screening	cPCR	5′-GATGATGTCTGAAGATATGAAACAAAT-3′ 5′-CTGCTCGTCTATTTTACTTCTTAAAGT-3′	409	95 °C for 2 min; 50 cycles: 95 °C for 15 s, 58 °C for 30 s and 72 °C For 30 s and a final extension 72 °C for 5 min	^28^
*Neorickettsia ristici* (16S rRNA gene) ER3-F ER2-R ER 3a-F ER2a-R	Screening	nPCR	5′-ATTTGAGAGTTTGATCCTGG-3′ 5′-GTTTTAAATGCAGTTCTTGG-3′ 5′-CTAGCGGTAGGCTTAAC-3′ 5′-CACACCTAACTTACGGG-3′	450	94 ºC for 5 min; 30 cycles: 94 ºC for 1 min, 60 ºC for 2 min and 72 ºC for 1 min, and a final extension 72 ºC for 7 min	^29^
*Bartonella* spp. (*nuoG* gene) F-Bart R-Bart Probe	Screening	qPCR	5′-CAATCTTCTTTTGCTTCACC-3′ 5′-TCAGGGCTTTATGTGAATAC-3′ TexasRed-5′-TTY GTCATTTGAACACG-3′ [BHQ2a-Q]-3′	83	95 °C for 3 min followed by 40 cycles at 95 °C for 10 s and 52.8 °C for 30 s	^30^
*Bartonella* spp. (*pap-31* gene) Pap31 1(s) Pap31 688 (as)	Characterization	cPCR	5′-GACTTCTGTTATCGCTTTGATTT-3′ 5′-CACCACCAGCAAMATAAGGCAT-3′	564	94 °C for 2 min, followed by 54 cycles of 94 °C for 15 s, 58 °C for 45 s, an extension at 72 °C for 45 s, and a final extension at 72 °C for 7 min	^31^
*Bartonella* spp. (*ftsZ* gene) ftsZF ftsZR	Characterization	cPCR	5′-CATATGGTTTTCATTACTGCYGGTATGG-3′ 5′-TTCTTCGCGAATACGATTAGCAGCTTC-3′	600	94 °C for 2 min, followed by 40 cycles of 94 °C for 30 s, 53 °C for 30 s, an extension at 72 °C for 15 s, and a final extension at 72 °C for 5 min	^32^
*Bartonella* spp. (*gltA* gene) CS443F CS1210R	Characterization	cPCR	5′-GCT ATG TCT GCA TTC TAT CA-3′ 5′-GAT CYT CAA TCA TTT CTT TCA-3′	750	94 °C for 5 min, followed by 35 cycles of 94 °C for 45 s, 61 °C for 45 s, an extension at 72 °C for 45 s, and a final extension at 72 °C for 7 min	^33^
Hemotropic *Mycoplasma* spp. (16S rRNA gene) HemoF1 HemoR2 HemoF2	Screening	snPCR	5′-AGAGTTTGATCCTGGCTCAG-3′ 5′-TACCTTGTTACGACTTAACT-3 5′-ATATTCCTACGGGAAGCAGC-3′	1107	95 °C for 5 min, followed by 35 cycles of 95 °C for 30 s, 57 °C for 30 s, an extension at 72 °C for 1 min, and a final extension at 72 °C for 10 min, for both rounds	^34^
*Hepatozoon* spp. (18S rRNA) HAM-1 HPF-2 4558 2733	Screening	nPCR	5′ GCCAGTAGT CATATGCTTGTC 3′ 5′ GACTTCTCCTTCGTCTAAG 3′ 5′ GCTAATACATGAGCAAAA TCTCAA 3′ 5′ CGGAATTAACCAGACAAAT 3′	1120	95 °C for 3 min, followed by 40 cycles of 95 °C for 1 min, 56 °C for 1 min, an extension at 72 °C for 1 min and 30 s, and a final extension at 72 °C for 7 min	^35,36^
Piroplasmids (18S rRNA gene) BTF1 BTR1 BTF2 BTR2	Screening	nPCR	5′-GGCTCATTACAACAGTTATAG-3′ 5′-CCCAAAGACTTTGATTTCTCTC-3′ 5′-CCGTGCTAATTGTAGGGCTAATAC-3′ 5′-GGACTACGACGGTATCTGATCG-3′	800	94 °C for 3 min, followed by 45 cycles of 94 °C for 30 seg, 58 °C for 20 seg, 72 °C for 30 seg, and a final extension at 72 °C for 7 min	^37^

*Ehrlichia canis* (Jaboticabal strain—maintained in DH82 cell culture), *Anaplasma phagocytophilum* (Webster strain) and *Neorickettsia risticii* (kindly provided by Professor John Stephen Dumler (Uniformed Services University of the Health Sciences, Bethesda, MD, USA) DNA were used as positive controls for Anaplasmataceae screening and characterization protocols. In the qPCR assays for *Bartonella* spp., serial dilutions were performed to construct standard curves with different concentrations (2.0 × 10^7^ to 2.0 × 10^0^ copies) of a plasmid encoding a fragment of the 83 bp nuoG gene of *Bartonella henselae* DNA (pIDTSMART; Integrated DNA Technologies). The number of plasmid copies was determined according to the formula (XG/μL DNA/ [Plasmid size (BP) × 660]) × 6, 22 × 10^23^ × plasmid copies/μL^25^. For hemotropic mycoplasmas, *Mycoplasma parvum* DNA from a naturally infected domestic pig was used^26^. DNA from *Hepatozoon* sp. detected in a naturally infected maned-wolf (*Chrysocyon brachyurus*) was used as positive control^27^. For Piroplasms screening, *Babesia vogeli* DNA (Jaboticabal strain) were used as a positive control. Ultra-pure sterile water (Life Technologies®, Carlsbad, CA, USA) was used as negative control in all PCR assays.

### Agarose gel electrophoresis, amplicon purification and Sanger sequencing

The results of cPCR, nPCR and semi-nPCR assays were visualized in 1% agarose gel stained by ethidium bromide solution and results were visualized using a UV transilluminator (ChemiDoc MP Imaging System, Bio Rad®). Positive samples were purified using ExoSAP-IT™ PCR Cleanup (ThermoFisher®) and sequenced. The sequencing was performed by Sanger method^28^ using ABI PRISM 3730 DNA Analyzer (Applied Biosystems) at Human Genome and Stem Cell Research Center, “Instituto de Biociências”, University of São Paulo (USP), Brazil.

### Phylogenetic analyses

Eletropherograms results were analyzed using Phred-Phrap software version 23, and the quality of each nucleotide sequence was checked for a score and considered of good quality when Phred score was higher than 20^29^. Consensus sequences obtained by the alignment of the forward and reverse sequences were constructed using the same software. The BLASTn program was used to analyze consensus sequences, aiming to browse and compare with sequences from the GenBank international database^30^. All sequences obtained in the present study were deposited in NCBI platform GenBank (https://www.ncbi.nlm.nih.gov/genbank/—Nucleotide) (Supplementary Information—Table SM1).

The obtained sequences were aligned with those retrieved from GenBank using MAFFT software, version 7^31^. For each studied agent and target gene, sequences were selected for phylogenetic inferences based on BLAST results and other studies performed in Brazil and other countries. For phylogenetic analyses, initially, the best model of evolution was selected by the program IQTREE (available at: http://iqtree.cibiv.univie.ac.at/), under the Akaike Information Criterion (AIC)^32^. Maximum likelihood (ML) analysis was performed with iqtree (available at: http://iqtree.cibiv.univie.ac.at/) and Bayesian analyses were performed using CIPRES gateway^33^. The phylogenetic tree edition and rooting (outgroup) were performed using the Treegraph 2.0 beta software^34^.

## Results

### DNA extraction and quality evaluation

Among the 49 ring-tailed coatis’ DNA blood samples, all were positive in the cPCR based on the *gapdh* gene and were included in subsequent analysis.

### PCR assays for *Anaplasma* spp

Seven (7/49—14.3%) coatis were positive in the nPCR for *Anaplasma* spp. based on the 16S rRNA gene. Six sequences (439-519 bp) were obtained and showed 99–100% identity (ID), 89–97% query-coverage (QC) and 0.0 E-value (EV) with *Anaplasma* sp. sequences previously detected in wild *Nasua nasua* from Mato Grosso do Sul State^20,21^ (GenBank accession numbers KY499184, KY499186-87, KY499192-95); and 100% ID, 88% QC and 0.0 EV with *Anaplasma* sp. detected in a rodent^35^. Four samples amplified for ITS—23S–5S (339–415 bp) and the obtained sequences showed 94.7% ID, 100% QC and 2e-114 EV with ‘*Candidatus* Anaplasma brasiliensis’ (MT267343-44) previously detected in anteaters from São Paulo state, southeastern Brazil^36^. Three samples amplified for *gltA* gene (569–599 bp) and the obtained sequences showed 83% ID, 90% QC, and 1e-14 EV with *Anaplasma* sp. detected in *Bos taurus* in Japan (JX445933).

**Figure 2:**
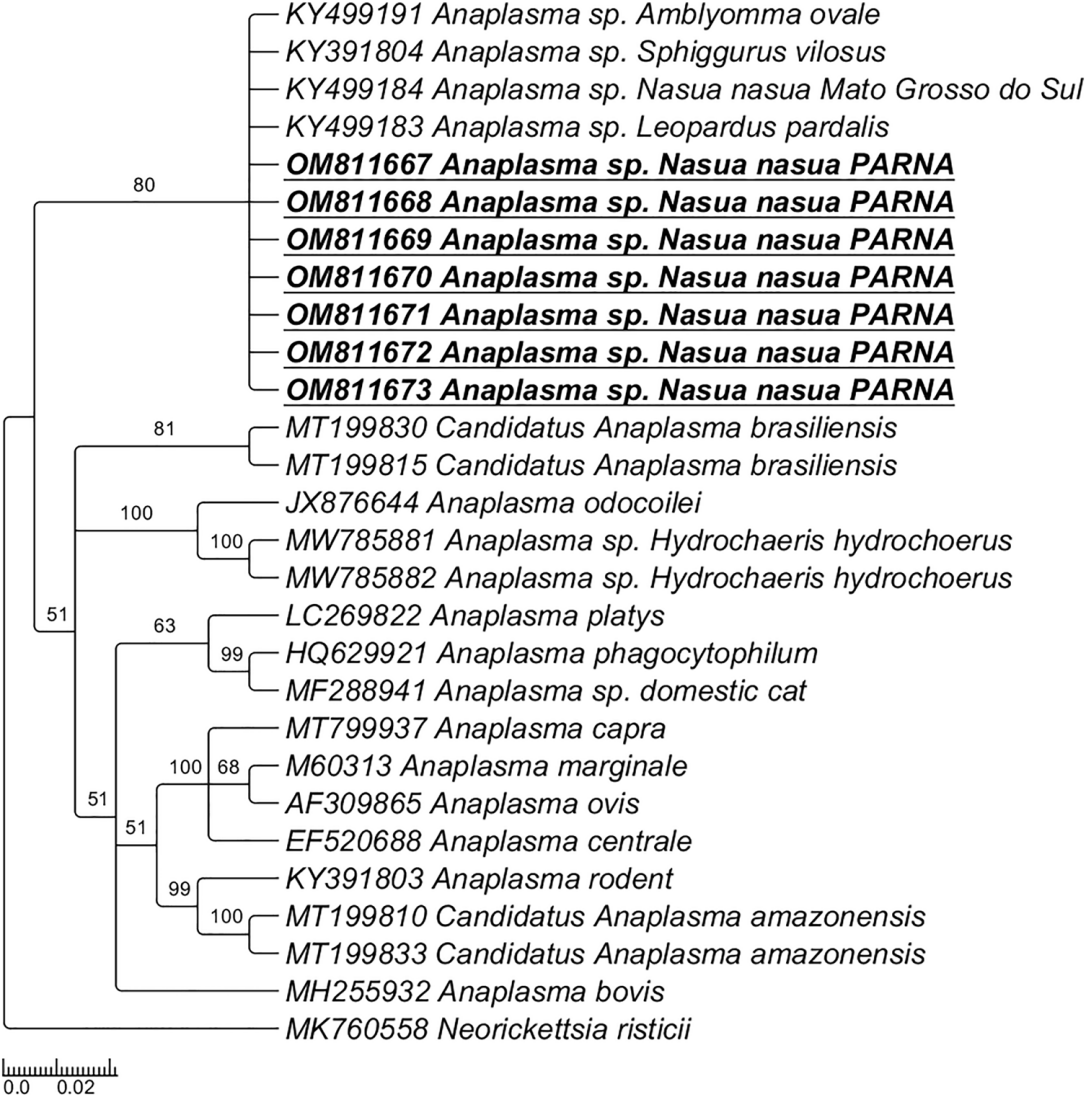
Phylogenetic trees inferred by the Bayesian method and based on 16S rRNA gene from *Anaplasma* spp. sequences obtained from blood from ring-tailed coatis (*Nasua nasua*) from PARNA. Sequences of *Anaplasma* sp. detected in the present study are highlighted in bold/underlined. *Neorickettsia risticii* was used as outgroup.

**Figure 3:**
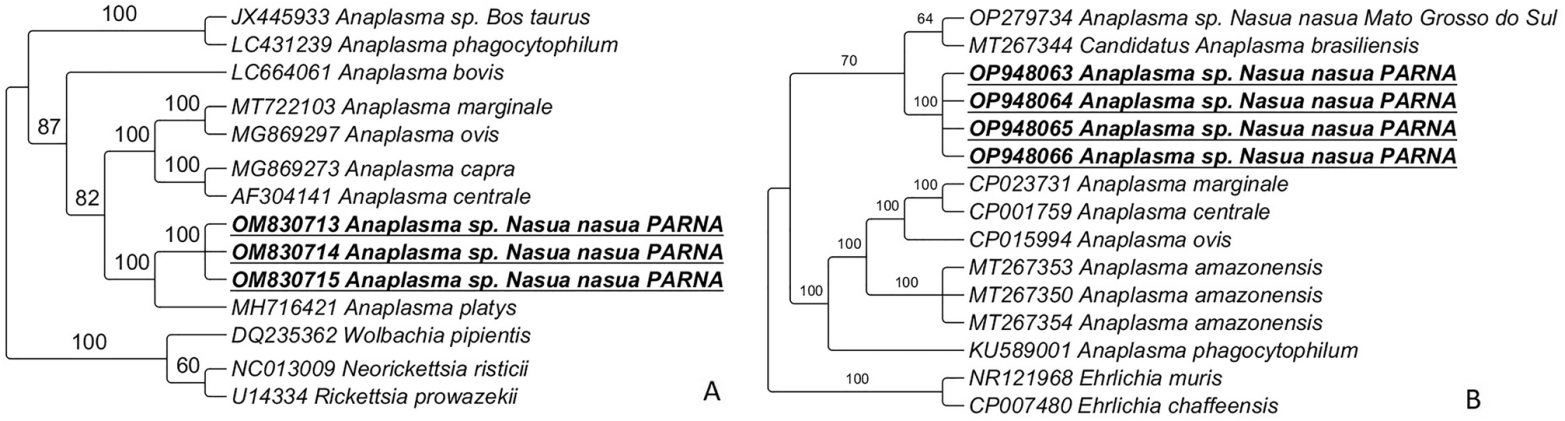
Phylogenetic trees inferred by the Bayesian method and based on *Anaplasma* spp. sequences obtained from blood from ring-tailed coatis (*Nasua nasua*) from PARNA. Sequences of *Anaplasma* sp. detected in the present study are highlighted in bold/underlined. (**A**) *gltA* gene; *N. risticii, Wolbachia pipientis* and *Rickettsia prowazekii* were used as outgroup; (**B**) ITS—23S–5S; *Ehrlichia muris* and *Ehrlichia chaffeensis* were used as outgroup.

Maximum likelihood (ML) analyses based on the 16S rRNA gene (519 bp) and TIM2 + I + G4 evolutionary model clustered the sequences detected in the present study in a large clade with other *Anaplasma* sp. sequences previously detected in wild animals from Brazil (Fig. 2). ML analyses based on the *gltA* gene (alignment of 655 bp and TPM3 + G4 as evolutionary model) (Fig. 3A) clustered the sequences detected in the present in a new clade, with the same origin of *Anaplasma platys*. ML analyses based on the ITS region (23S–5S) (alignment of 420 bp and K3P + G4 as evolutionary model) (Fig. 3B) clustered the obtained sequences in a new clade with the same origin of ‘*Candidatus* Anaplasma brasiliensis’.

### PCR assay for *Hepatozoon* spp

Three (3/49—6%) coatis were positive in the nPCR for *Hepatozoon* spp. based on the 18S rRNA gene. The three obtained sequences (945, 985 and 1031 bp) showed 100% ID, 100% QC, and 0.0 EV with *Hepatozoon procyonis* detected in coatis from Brazil (GenBank accession numbers MF685386-MF685410 and MW862003-MW862006).

Phylogenetic analyses (ML method and GTR as evolutionary model) based on an alignment of 1017 bp of the 18S rRNA gene clustered all three sequences detected in the present study in a high supported clade (100% of bootstrap) with *Hepatozoon procyonis* sequences previously detected in ring-tailed coatis from other Brazilian states (Figure SM2—Supplementary file).

### PCR assay for hemotropic *Mycoplasma* spp

Forty-two (42/49—85.7%) ring-tailed coatis samples were positive in the snPCR for hemotropic *Mycoplasma* spp. based on the 16S rRNA gene. Eight samples were randomly selected for sequencing (892–941 bp). All samples presented 99.8–100% ID, 100% QC, and 0.0 EV with ‘*Candidatus* Mycoplasma haematonasua’ and hemotropic *Mycoplasma* sp. previously detected in coatis from Mato Grosso do Sul state and Paraná state, Brazil. ML analyses (alignment of 941 bp and TN + I + G4) clustered the sequences detected in the present study in a large clade with other sequences previously detected in coatis from Brazil, with 100% of clade support (Figure SM3—Supplementary file).

### PCR assays for *Bartonella* spp

**Figure 4:**
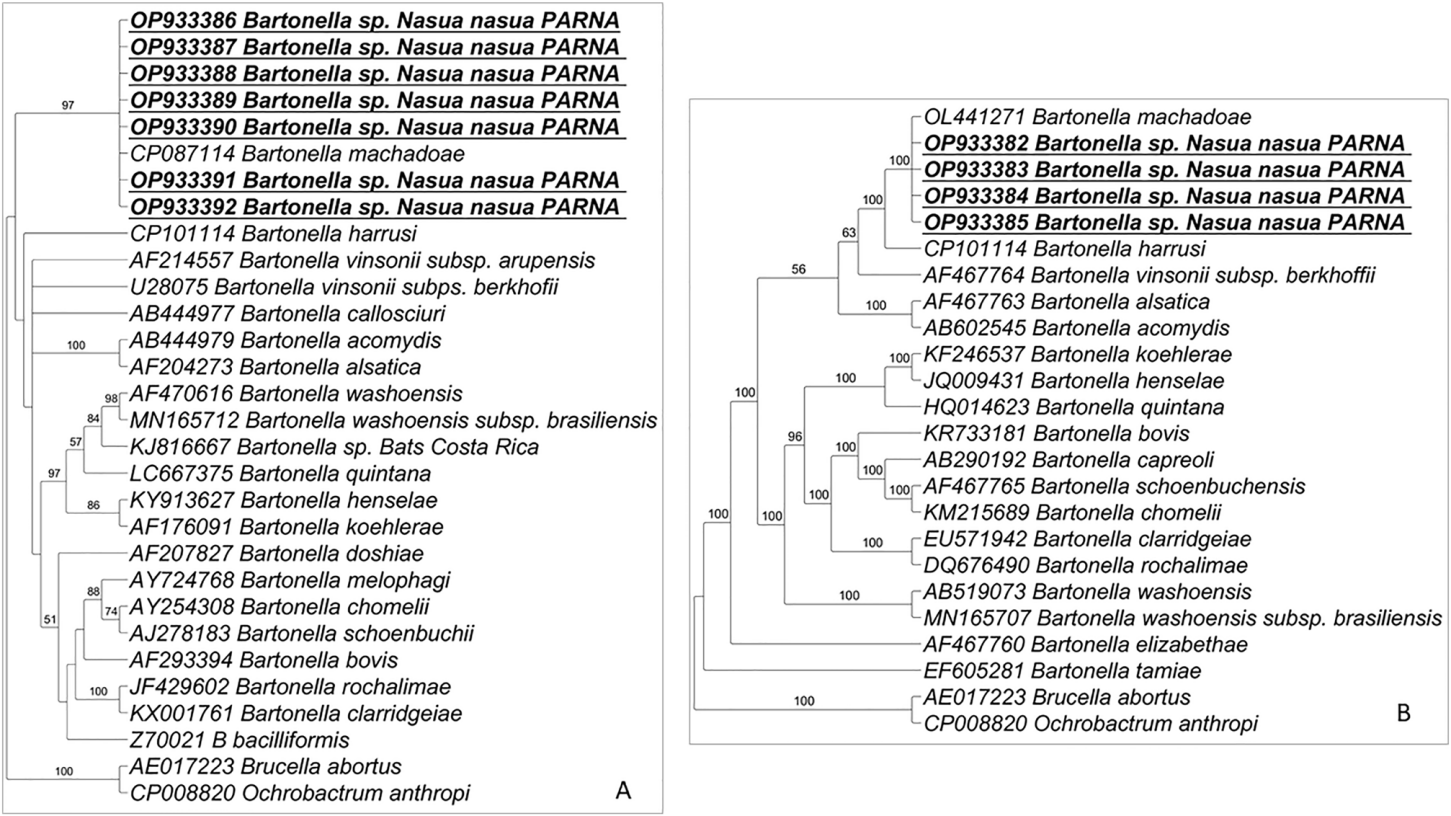
Phylogenetic trees based on the Maximum likelihood method from *Bartonella* spp. sequences obtained from blood from ring-tailed coatis (*Nasua nasua*) from PARNA. Sequences of *Bartonella* sp. detected in the present study are highlighted in bold/underlined (**A**) *gltA* gene; *Brucella abortus* and *Ochrobactrum anthropi* were used as outgroup; (**B**) *ftsZ* gene; *Brucella abortus* and *Ochrobactrum anthropi* were used as outgroup.

Twelve samples (12/49—24.5%) showed positivity in qPCR assays for *Bartonella* spp. based on the *nuoG* gene. The number of copies of *Bartonella*-*nuoG* fragment/μL ranged from 1.38 × 10^1^ to 5.10 × 10^1^. The mean values of efficiency, correlation coefficient, and y-intercept of qPCR reactions mean were 103.4%, 0.920, and 36.031, respectively. Five out of 12 qPCR-positive samples for *Bartonella* also showed positivity in a PCR assay based on the *pap-31* gene. The obtained sequences ranged from 382 to 532 bp and showed 99.06–100% ID, 100% QC, 0.0 E-value and with *Bartonella machadoae*^37^. Seven out of 12 qPCR-positive samples for *Bartonella* also showed positivity in a PCR assay based on the *gltA* gene, with sequences ranging from 401 to 582 bp and showing 99.83–100% ID, 100% QC and 0.0 EC with *Bartonella machadoae*. Four out of 12 qPCR-positive samples for *Bartonella* also showed positivity in a PCR assay based on the *ftsZ* gene, with sequences ranging from 505 to 549 bp and showing 100% ID, 100% QC, and 0.0 EV and with *Bartonella machadoae*. Bayesian phylogenetic analyses based on both the *gltA* gene (alignment of 578 bp and TIM2 + G as evolutionary model) and the *ftsZ* gene (alignment of 538 bp and HKY + I + G4 as evolutionary model) clustered all sequences detected in the present study in a clade with *Bartonella machadoae* (Fig. 4).

### PCR assays for* Ehrlichia *spp.*, Neorickettsia risticii and* Piroplasmids

None of the 49 ring-tailed coatis’ DNA blood samples were positive in the molecular assays for *Ehrlichia* spp., *Neorickettsia risticii* and piroplasmids.

### Co-infection by vector-borne agents

Only four animals were negative for all the vector-borne agents tested. None of the examined ring-tailed coatis were positive for all tested agents. The most co-infection observed was from bacterial agents, in first place with hemotropic *Mycoplasma* sp. and *Bartonella* sp. (9 animals) and in second place *Anaplasma* sp. with hemotropic *Mycoplasma* sp. (6 animals). Only two animals presented co-infections by three agents (*Bartonella* sp., *Anaplasma* sp. and hemotropic *Mycoplasma* sp.) All information about positive animals for the investigated agents regarding sampling date, sex and co-infections are shown in Table SM2—Supplementary file). All co-infections among the investigated agents are presented in a Venn Diagram (Figure SM4—Supplementary file).

## Discussion

Herein, wild ring-tailed coatis were found to be infected with a potential novel genotype of *Anaplasma* sp., related to genotypes previously detected in ectoparasites (e.g. *Amblyomma sculptum, Amblyomma ovale*) and wild animals (e.g. *Sphiggurus villosus*, *Leopardus pardalis*)^20^, and phylogenetically related to ‘*Candidatus* Anaplasma brasiliensis’^36^. *Anaplasma* spp. belong to Anaplasmataceae family and Order Rickettsiales and are obligate intracellular α-proteobacteria that can cause important diseases in humans and animals^38,39^. Representatives of Anaplasmataceae agents have been detected in several mammal species, and wild and domestic carnivores are considered an important source of these agents, especially in anthropized areas, where a close contact among domestic/wild animals and humans can occur^39,40^. In the USA, raccoons (*Procyon lotor*) play a role as reservoirs for *Anaplasma phagocytophilum*, a zoonotic agent^39,41,42^. When it comes to coatis, Sousa et al.^21^ detected an *Ehrlichia* 16S rDNA genotype phylogenetically related to multiple *Ehrlichia* spp. previously detected in ticks and wild rodents from Brazil. Additionally, two different *Anaplasma* 16S rRNA genotypes were detected in ring-tailed coatis from the Brazilian Pantanal: while one was positioned in a clade related to *A. phagocytophilum,* the other one clustered with *A*. *bovis*. Also, *Ehrlichia/Anaplasma* 16S rDNA was detected in ring-tailed coatis from the same area (PARNA) where the present study was conducted, but the authors were not able to sequence the positive sample^13^. In the present study, phylogenetic inferences based on the *gltA* gene positioned the *Anaplasma* sp. Detected in coatis into a new clade, with the same origin of *Anaplasma platys*. In the ITS (23S–5S)-based phylogeny, the *Anaplasma* genotypes detected showed to be closely related to *‘Candidatus* Anaplasma brasiliensis’. These findings suggest that a novel *Anaplasma* species, yet to be isolated and characterize, occurs in ring-tailed coatis from Brazil.

In the present study, a lower occurrence for *H. procyonis* was found when compared to those previously reported in ring-tailed coatis from other Brazilian regions. The genus *Hepatozoon* comprise apicomplexan hemogregarinids with a heteroxene life cycle, that infects a wide range of vertebrates, including mammals, reptiles, birds and amphibians^43^. Up to now, the only *Hepatozoon* species known to infect Procyonidae mammals is *H. procyonis*, that was first described in 1961 infecting monocytes of raccoons (*P. lotor*) in the USA^44^. In Brazil, occurrence rates of 42% (13/42), 25% (21/93), 32% (69/262), and 68% (112/165) were reported in ring-tailed coatis from the Pantanal wetlands of Mato Grosso do Sul state, São Paulo state, Minas Gerais state, and peri-urban forested areas (Cerrado biome) in Mato Grosso do Sul states, respectively^15–18^. In the present study, only 3/49 (6%) samples tested positive for *Hepatozoon* sp. This lower prevalence might not be related to the applied molecular technique, since a nested-PCR based on the 18S rRNA was used, which showed to be very sensitive in the detection of *H. procyonis* with fluctuating parasitemia^18^. Considering that the vector for *H. procyonis* is not known yet, the lower prevalence found herein might be due to the tick fauna presented (and consequently vector competence) in the studied area when compared to the other studies. *Amblyomma* spp. larvae, and adults of *Haemaphysalis* *juxtakochi, Amblyomma brasiliense*, *Amblyomma coelebs* and *Amblyomma ovale* have been reported in coatis from PARNA^45^*.* When compared to other studies conducted in Mato Grosso do Sul^14,15^, São Paulo^46,47^, and Minas Gerais^17^ states, where high occurrence of *H. procyonis* has been reported in the sampled ring-tailed coatis, *A. sculptum* ticks were not found parasitizing the animals sampled herein^45^. The role of *A. sculptum* and other tick species in the transmission of *H. procyonis* should be further evaluated in the future.

The most prevalent VBA detected in ring-tailed coatis from PARNA Iguaçu was *Mycoplasma* sp. closely related to *‘Candidatus* Mycoplasma haematonasua’, which was found in 85.7% of the studied animals. Hemoplasmas (hemotropic mycoplasmas) are obligate epi-erythrocytic bacteria that infect a wide variety of mammalian hosts. Infections with this agent can induce anemia and acute hemolysis and clinical signs, such as anorexia, lethargy, dehydration, weight loss and, in some cases, sudden death in domestic animals^48^, while the infection appears to be subclinical in wildlife^49^. In animals of the Procyonidae family, hemotropic *Mycoplasma* sp. 16S rRNA genotypes phylogenetically related to “*Candidatus* Mycoplasma erythrodidelphis”, “*Candidatus* Mycoplasma haemozalophi”, *Candidatus* Mycoplasma haemominutum” and “*Candidatus* Mycoplasma haemolamae” were detected in 59/95 (62.1%) raccoons in the USA^50^. In Brazil, Sousa et al.^12^ found 24/31 (77.4%) wild ring-tailed coatis in the Brazilian Pantanal positive for hemotropic *Mycoplasma* sp. in a PCR based on the 16S rRNA gene. Interestingly, authors detected two different genotypes: one closely related to hemotropic *Mycoplasma* sp. detected in raccoons from the USA, and another one closely related to *Mycoplasma haemofelis*. More recently, Collere et al.^13^ detected hemotropic *Mycoplasma* sp. in 44.5% (8/18) coatis in the same area (PARNA) where the present study was conducted. Based on two molecular markers, namely 16S rRNA and 23S rRNA, a putative novel hemoplasma, ‘*Candidatus* Mycoplasma haematonasua’, was proposed. It seems that hemoplasmas are the most prevalent VBA detected in Procyonidae animals from the Americas. Although some hemotropic *Mycoplasma* species (e.g. *Mycoplasma suis, Mycoplasma haemocanis, Mycoplasma haemofelis*)^51^ can cause moderate to severe disease in their hosts, the pathogenicity of ‘*Candidatus* M. haematonasua’ is not yet known, requiring more studies, especially in animals infected with more than one VBA. The main route of transmission of hemoplasmas seems to be through blood-sucking arthropods (e.g. fleas, ticks, etc.)^48,52^. However, few experimental studies have been carried out in this sense, since such agents have not been yet cultivated in vitro^48^. Other routes of transmission have also been pointed out, such as vertical, blood transfusion) ^53,54^ and through aggressive interactions among cats^55^ and wild rodents (*Gerbillus andersoni*)^56^. Based on multilayer network analysis, Alcantara et al.^57^ recently demonstrated the lack of association among ectoparasites, non-hematophagous bats and hemoplasmas, suggesting that ectoparasites may not play an important role in the transmission of hemotropic mycoplasmas among bats. Considering these putative alternative transmission routes, Sousa et al.^12^ hypothesized that the gregarious behavior of coatis associated to the fact that these mammals tend to form large groups^1^ might contributed for a higher incidence of hemotropic *Mycoplasma* spp. among this Procyonidae species.

The present study shows, for the first time, the molecular detection of *Bartonella* sp. in blood samples from ring-tailed coatis from Brazil. The genus *Bartonella* (Rhizobiales: Bartonellaceae) comprises Gram-negative, facultative intracellular alpha-proteobacteria, which have been identified in a wide variety of mammals, including humans^58–60^. The transmission of bartonellae has been mainly associated with hematophagous arthropod vectors^61^. Up to now, *Bartonella* spp. has only been molecularly detected in raccoons from the USA. Hwang and Gottdenker^62^, when studying free-living raccoons from Georgia, USA, detected 16 positive samples (43%—16/37) in a nested-PCR based on the 16S–23SrRNA intergenic spacer region (ITS). Thirteen positive samples were sequenced and 12 samples were identified as *B. henselae* and one identified as *B. koehlerae*^62^*.* Bai et al.^63^, when analyzing 186 spleen samples from raccoons during the enhanced rabies surveillance in Colorado, USA, obtained 14 samples positive in a PCR for *Bartonella* spp. based on the ITS (16S-23S) region. Sequencing results revealed that 11 raccoons were infected with *B. rochalimae* and three with *Bartonella vinsonii* subsp. *berkhoffii* ^63^, recently reclassified as *Bartonella berkhoffii* by Whole Genome Sequencing Analysis^64^.

When analyzing coatis from the Pantanal wetlands in Mato Grosso do Sul state, Brazil, Sousa et al.^65^ failed to detect DNA from *Bartonella* sp. In blood samples from 31 sampled coatis. In the present study, 24.5% (12/49) showed to be positive in a qPCR based on the *nuoG* gene. By sequencing three molecular markers (*gltA, pap-31*, and *ftsZ), B. machadoae* was identified in ring-tailed coatis from PARNA. *Bartonella machadoae* has been recently isolated and described from wild rodents from the Pantanal wetlands, Mato Grosso do Sul state, central-western Brazil, and showed to be strictly related to species belonging to *B. vinsonii* complex^37^. The zoonotic potential is yet unknown, and further studies should be carried out, since *Bartonella* species belonging to *B. vinsonii* complex are associated with clinical manifestations in humans in different regions of the world^37^. Fleas have been suggested as putative vector for *Bartonellla* among rodents in Brazil^65,66^. Indeed, *Bartonella* sp. has been molecularly detected in *Polygenis bohlsi bolshi* fleas collected from rodents in the Pantanal wetlands biome, Mato Grosso do Sul state, central-western Brazil^65^, as well as in *Polygenis occidentalis, Polygenis platensis*, and *Craneopsylla minerva* from Pampa grasslands and Atlantic rainforest biomes, in the State of Rio Grande do Sul, southern Brazil^66^*.* Recently, *B. machadoae* has been molecularly detected in *Amblyomma sculptum* nymphs collected from wild boars (*Sus scrofa*), which showed to be negative for *Bartonella* spp. in a qPCR based on the *nuoG* gene, in southeastern Brazil. According to the authors, *A. sculptum* nymphs may have acquired the *Bartonella* infection when fed as larvae in a mammal (most probably rodents), and transestadially perpetuate the infection through the nymph stage. It this is true, *A. sculptum* may play a role in the transmission of *B. machadoae*^67^. The present study expands the host specificity range of *B. machadoae*, which has been isolated only from rodents until the present moment^37^.

A small percentage (8.1%) of animals were negative for the screened VBA in the present study. The most prevalent co-infection observed was from bacterial VBA agents, in first place (18.3%) with hemotropic *Mycoplasma* sp. and *Bartonella* sp., and *Anaplasma* sp. and hemotropic *Mycoplasma* sp. in second place (12.2%) Only two animals (4%) presented co-infections by three VBA (*Bartonella* sp., *Anaplasma* sp. and hemotropic *Mycoplasma* sp.). Although VBA infections seem to be associated with subclinical diseases in wild animals^18,49^, VBA may play a role as potential opportunistic pathogens in immunocompromised animals, in stressful situations or in coinfections. For instance, Moraes et al.^23^ demonstrated that the infection by filarial nematodes in ring-tailed coatis had no effects on the body condition and serum biochemistry parameters of the evaluated coatis, albeit eosinophilia was observed. Also, Perles et al.^18^ showed that the *H. procyonis* infection seems not to significantly influence clinical and hematological parameters. Co-infections may play an important role in these infections. For instance, Alic et al.^68^ described a fatal case caused by coinfection by *Leptospira* spp. and *H. canis* in a red fox cub (*Vulpes vulpes*) in Saravejo, Bosnia and Herzegovina. Also, Silveira et al.^69^ reported a fatal case of co-infection by *Rangelia vitalii,* *Hepatozoon* sp., *Leishmania* sp., and *Entamoeba* and helminths (*Ancylostoma caninum, Ancylostoma braziliensis*, *Molineus* sp., *Trichuris* sp. and *Spirometra* sp.) in the threatened maned-wolf (*Chrysocyon brachyurus*) from Brazil.

Our results highlight the importance of assessing the health of wild animals in one of the largest remaining areas of Atlantic forest in Brazil. Our observations also reveal the needs to deepen and expanding this type of studies for other mammals and possible vectors inventories. The coati, besides common, play an important role in food chain, being also the symbol of the company that provides tourism care and probably the only wild mammal observed during the INP tourist visit. Since the zoonotic potential and transmission routes of the detected VBA are not yet known, VBA surveillance in this area is much needed. Future studies are necessary to understand the pathogenicity of the detected agents to coatis as well as their zoonotic potential. Also, new studies combining other methodologies with molecular detection, such as isolation, blood smears to investigate the target host cells, and health analyses (e.g. hematological and biochemical analyses) would provide a better approach on the surveillance and understanding of the pathogenicity of such agents.

## Conclusions

Ring-tailed coatis from Iguaçu National Park were found infected with different vector-borne agents. The most prevalent VBA was *Mycoplasma* sp. closely related to ‘*Candidatus* Mycoplasma haematonasua’, followed by *Bartonella* sp*.*, *Anaplasma* sp., and *Hepatozoon procyonis*. A putative novel *Anaplasma* species, yet to be isolated and characterized, occurs in ring-tailed coatis from Iguaçu National Park. This is the first molecular detection of *B. machadoae* in coatis. Co-infections by *Mycoplasma* sp., *Bartonella* sp. and *Anaplasma* sp. are reported for the first time in coatis.

## Supplementary Information

Supplementary Information.

## Data Availability

The datasets generated and analysed during the current study are available in the NCBI—GenBank—Nucleotide platform (https://www.ncbi.nlm.nih.gov/genbank/) and can be accessed through accession numbers: ***Anaplasma***** sp.** (16SrRNA OM811667—OM811673), (ITS OP948063–OP948066), (*gltA* OM830713—OM830715); ***Hepatozoon***** sp.** (18SrRNA OM812694—OM812696), ***Mycoplasma***** sp.** (16SrRNA OP795503—OP795509), ***Bartonella***** sp.** (*pap31* OP910252–OP910256), (*ftsZ* OP93382–OP933385), (*gltA* OP933386–OP933392).
