# Supplementary material for: Co-infection by multiple vector-borne agents in wild ring-tailed coatis (Nasua nasua) from Iguaçu National Park, southern Brazil
Source: Sci Rep. 2023 Feb 1;13:1828. doi: 10.1038/s41598-023-29090-1 (PMC9892030; doi:10.1038/s41598-023-29090-1)
Supplement: Supplementary file 1 — Supplementary Information. [file 41598_2023_29090_MOESM1_ESM.docx]

**Co-infection by multiple vector-borne agents in wild ring-tailed coatis (*Nasua nasua*) from Iguaçu National Park, southern Brazil**

Perles, L.^1^; Moraes, M.F..^2^; Xavier da Silva, M.^3^; Vieira, R.F.C.^4,5,6^; Machado, R.Z.^1^; Lux Hoppe, E.G. ^2^; André, M.R.^1*^

^1^ Vector-Borne Bioagents Laboratory (VBBL), Department of Pathology, Reproduction and One Health, School of Agricultural and Veterinarian Sciences, São Paulo State University (Unesp), Jaboticabal, SP, Brazil

^2^ Laboratory of Parasitic Diseases (LabEPar), Department of Pathology, Reproduction and One Health, School of Agricultural and Veterinarian Sciences, São Paulo State University (Unesp), Jaboticabal, SP, Brazil

^3^ Iguaçu Carnivore Project, Iguaçu National Park, BR-469, Km 22.5, Foz do Iguaçu, Paraná 85851-970, Brazil

^4^ Vector-Borne Diseases Laboratory, Department of Veterinary Medicine, Universidade Federal do Paraná – UFPR, Curitiba, Brazil

^5^ Department of Public Health Sciences, University of North Carolina at Charlotte, Charlotte, USA.

^6^ Center for Computational Intelligence to Predict Health and Environmental Risks (CIPHER), University of North Carolina at Charlotte, Charlotte, USA.

***Corresponding author:** Prof. Dr. Marcos Rogério André, Vector-Borne Bioagents Laboratory (VBBL), Department of Pathology, Reproduction and One Health, School of Agricultural and Veterinarian Sciences, São Paulo State University (Unesp), Via de Acesso Prof. Paulo Donato Castellane, s/n, Zona Rural, CEP: 14884-900, Jaboticabal, São Paulo, Brazil. Phone: +55 (16) 3209-7302 Fax: +55 (16) 3202-4275. e-mail: mr.andre@unesp.br

**Table SM1.** GenBank accession number positive and sequenced samples according to agent and target gene. All sequences are available at NCBI platform <https://www.ncbi.nlm.nih.gov/genbank/>.

| ***Anaplasma* sp. sequences GenBank accession numbers** | | | |
| --- | --- | --- | --- |
| ID | *16S rRNA* | *ITS* | *gltA* |
| 9 | OM811667 | OP948063 | OM830713 |
| 49 | OM811668 | N/A | N/A |
| 56 | OM811669 | N/A | N/A |
| 60 | OM811670 | OP948064 | OM830714 |
| 68 | OM811671 | OP948065 | OM830715 |
| 70 | OM811672 | OP948066 | N/A |
| 71 | OM811673 | N/A | N/A |
| ***Hepatozoon procyonis* sequences GenBank accession numbers** | | | |
| ID | *18SrRNA* | | |
| 8 | OM812694 | | |
| 32 | OM812695 | | |
| 76 | OM812696 | | |
| **Hemotropic *Mycoplasma* sp. sequences GenBank accession numbers** | | | |
| ID | 16SrRNA | | |
| 26 | OP795503 | | |
| 29 | OP795502 | | |
| 50 | OP795504 | | |
| 52 | OP795505 | | |
| 53 | OP795506 | | |
| 60 | OP795507 | | |
| 71 | OP795508 | | |
| 77 | OP795509 | | |
| ***Bartonella* sp. sequences GenBank accession numbers** | | | |
| ID | *pap31* | *ftsZ* | *gltA* |
| 24 | N/A | N/A | OP933388 |
| 25 | N/A | OP933382 | OP933390 |
| 44 | OP910256 | OP933383 | OP933392 |
| 46 | N/A | OP933384 | N/A |
| 52 | OP910254 | N/A | OP933387 |
| 53 | OP910253 | OP933385 | N/A |
| 55 | N/A | N/A | N/A |
| 56 | N/A | N/A | N/A |
| 68 | OP910255 | N/A | OP933389 |
| 69 | N/A | N/A | OP933386 |
| 70 | N/A | N/A | N/A |
| 77 | OP910252 | N/A | OP933391 |

N/A: Non amplified

**Table SM2.** Identification of coatis sampled at Parque Estadual do Iguaçu, Paraná, Brazil, between 2016 and 2017, regarding identification number, collection data, sex and molecular positivity for vector-borne agents. Results obtained in the PCR assays for *Ehrlichia* sp., piroplasmids and *Neorickettsia risticii* are not shown since all animals tested negative.

| **ID** | **Collection data** | **Sex** | ***Hepatozoon* sp.** | ***Anaplasma* sp.** | ***Bartonella* sp.** | **Hemotropic *Mycoplasma* sp.** |
| --- | --- | --- | --- | --- | --- | --- |
| 2 | 03/11/2016 | M^*^ | - | - | - | Positive |
| 8 | 15/11/2016 | M | Positive | - | - | Positive |
| 9 | 17/11/2016 | F^**^ | - | Positive | - | Positive |
| 11 | 17/11/2016 | F | - | - | - | Positive |
| 15 | 18/11/2016 | M | - | - | - | Positive |
| 16 | 18/11/2016 | F | - | - | - | Positive |
| 21 | 14/12/2016 | F | - | - | - | Positive |
| 23 | 14/12/2016 | F | - | - | - | - |
| 24 | 05/02/2017 | F | - | - | Positive | Positive |
| 25 | 15/03/2017 | F | - | - | Positive | - |
| 26 | 12/03/2017 | M | - | - | - | Positive |
| 27 | 12/03/2017 | M | - | - | - | Positive |
| 29 | 12/03/2017 | M | - | - | - | Positive |
| 31 | 29/03/2017 | M | - | - | - | Positive |
| 32 | 29/03/2017 | F | Positive | - | - | Positive |
| 34 | 29/03/2017 | F | - | - | - | Positive |
| 35 | 29/03/2017 | M | - | - | - | - |
| 36 | 05/09/2017 | M | - | - | - | Positive |
| 37 | 05/09/2017 | F | - | - | - | Positive |
| 38 | 05/09/2017 | M | - | - | - | Positive |
| 39 | 05/09/2017 | F | - | - | - | - |
| 40 | 05/09/2017 | M | - | - | - | Positive |
| 41 | 05/09/2017 | F | - | - | - | Positive |
| 42 | 05/09/2017 | F | - | - | - | Positive |
| 43 | 05/09/2017 | F | - | - | - | Positive |
| 44 | 06/09/2017 | F | - | - | Positive | Positive |
| 45 | 07/09/2017 | F | - | - | - | Positive |
| 46 | 07/09/2017 | M | - | - | Positive | Positive |
| 48 | 08/09/2017 | M | - | - | - | Positive |
| 49 | 08/09/2017 | M | - | Positive | - | Positive |
| 50 | 08/09/2017 | M | - | - | - | Positive |
| 52 | 08/09/2017 | F | - | - | Positive | Positive |
| 53 | 08/09/2017 | M | - | - | Positive | Positive |
| 54 | 09/09/2017 | F | - | - | - | Positive |
| 55 | 10/09/2017 | F | - | - | Positive | - |
| 56 | 10/09/2017 | M | - | Positive | Positive | - |
| 57 | 10/09/2017 | M | - | - | - | Positive |
| 58 | 10/09/2017 | M | - | - | - | Positive |
| 60 | 11/09/2017 | F | - | Positive | - | Positive |
| 62 | 12/09/2017 | F | - | - | - | Positive |
| 63 | 12/09/2017 | F | - | - | - | Positive |
| 65 | 13/09/2016 | F | - | - | - | Positive |
| 68 | 13/09/2016 | F | - | - | Positive | Positive |
| 69 | 15/09/2016 | M | - | Positive | Positive | Positive |
| 70 | 15/09/2016 | F | - | Positive | Positive | Positive |
| 71 | 15/09/2016 | F | - | Positive | - | Positive |
| 73 | 23/09/2017 | M | - | - | - | Positive |
| 76 | 27/09/2017 | F | Positive | - | - | Positive |
| 77 | 27/09/2017 | F | - | - | Positive | Positive |

* Male; **Female; - Negative

**Figure SM1.** Flow chart of the molecular assays (screening and characterization) for vector-borne agents performed in the present study.


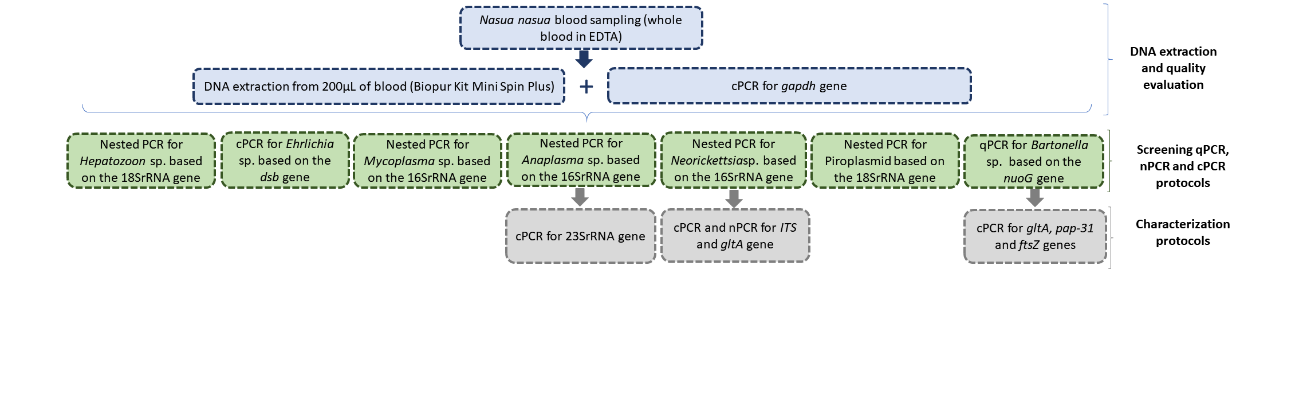


**Figure SM2.** Maximum likelihood phylogenetic tree based on *Hepatozoon* spp. 18S rRNA sequences. Numbers at nodes correspond to bootstrap. Accession numbers are indicated in the sequences. Sequences of *Hepatozoon procyonis* detected in the present study are highlighted in bold/underlined. *Dactylosoma ranarum* was used as outgroup.

**Figure SM3.** Bayesian phylogenetic tree based on *Mycoplasma* spp. 16S rRNA sequences. Numbers at nodes correspond to bootstrap. Accession numbers are indicated in the sequences. Sequences of hemotropic *Mycoplasma* sp*.* detected in the present study are highlighted in bold/underlined. *Mycoplasma pneumoniae* and *Bacillus subtilis* were used as outgroup.

**
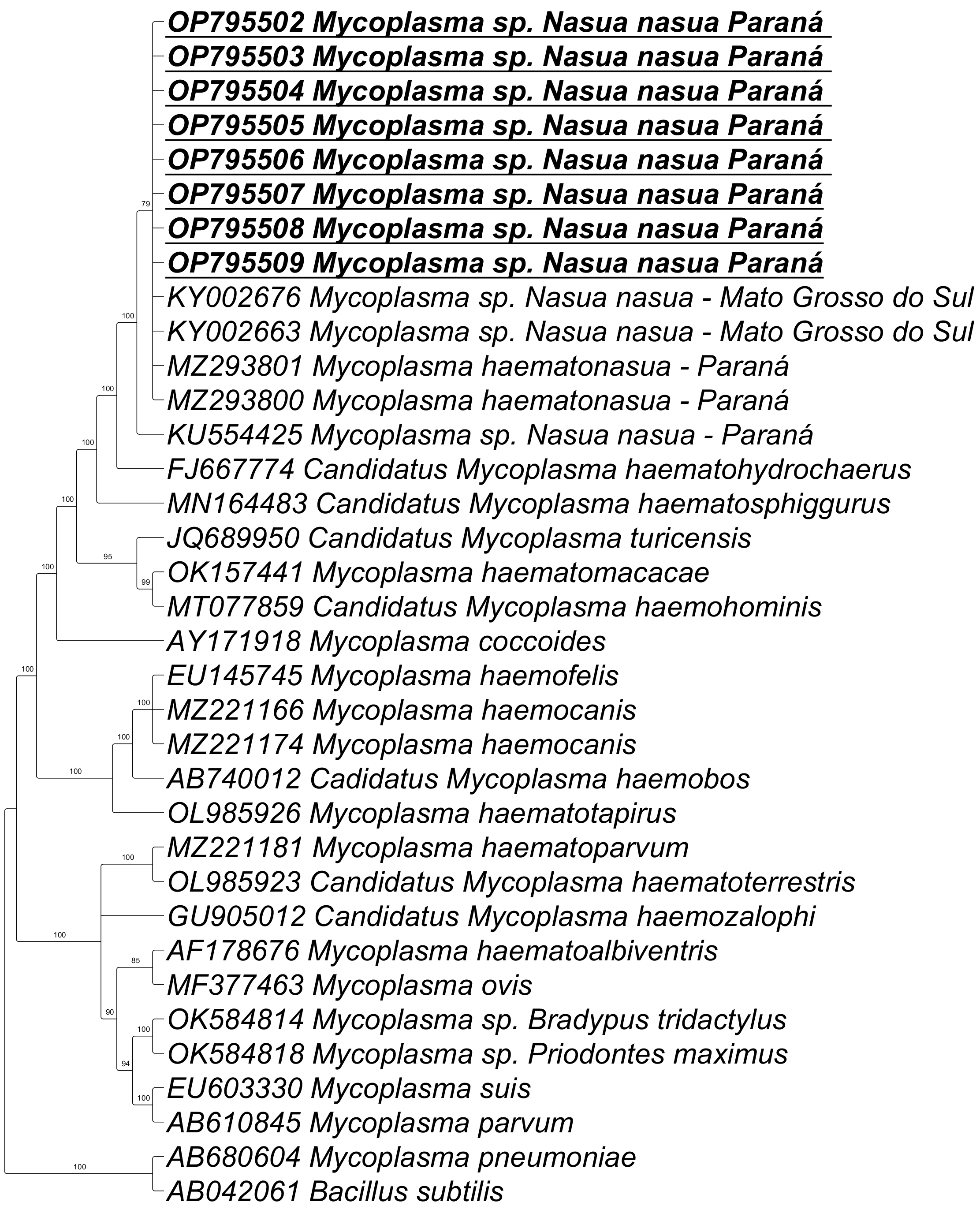
**

**Figure SM4.** Venn Diagram showing all interactions of the detected vector-borne pathogens in blood samples from coatis (*Nasua nasua*) in Parque Nacional do Iguaçu, Foz do Iguaçu, Brazil.

**
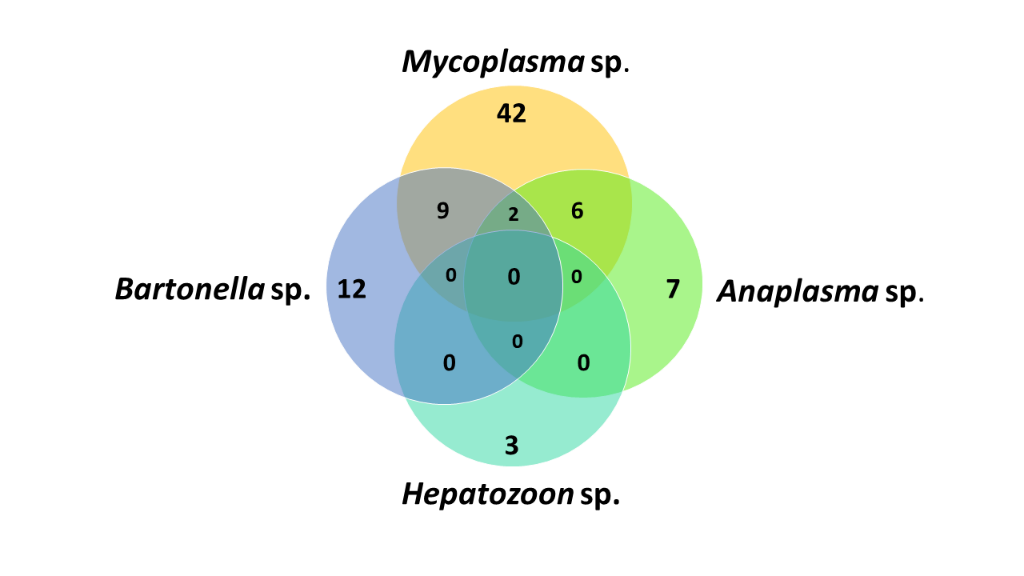
**
